# Occupational demands associated with rotator cuff disease surgery in the UK Biobank

**DOI:** 10.5271/sjweh.4062

**Published:** 2022-12-30

**Authors:** Elizabeth L Yanik, Jay D Keener, Martin J Stevens, Karen E Walker-Bone, Ann Marie Dale, Yinjiao Ma, Graham A Colditz, Rick W Wright, Nancy L Saccone, Nitin B Jain, Bradley A Evanoff

**Affiliations:** 1 Washington University School of Medicine, St. Louis, MO, USA; 2 Vanderbilt University School of Medicine, Nashville, TN, USA; 3 University of Southampton, Southampton, UK

**Keywords:** job exposure matrix, occupational health

## Abstract

**Objectives:**

Physically-demanding occupations may increase rotator cuff disease (RCD) risk and need for surgery. We linked a job-exposure matrix (JEM) to the UK Biobank cohort study to measure physical occupational exposures and estimate associations with RCD surgery.

**Methods:**

Jobs and UK Standard Occupational Classification (SOC) codes were recorded during the UK Biobank verbal interview. Lifetime job histories were captured through a web-based survey. UK SOC codes were linked to a JEM based on the US O*NET database. O*NET-based scores [static strength, dynamic strength, general physical activities, handling/moving objects (range=1–7), time spent using hands, whole body vibration, and cramped/awkward positions (range=1–5)] were assigned to jobs. RCD surgeries were identified through linked national hospital inpatient records. Multivariable Cox regression was used to calculate hazard ratios (HR) as estimates of associations with RCD surgery. Among those with lifetime job histories, associations were estimated for duration of time with greatest exposure (top quartile of exposure).

**Results:**

Of 277 808 people reporting jobs, 1997 (0.7%) had an inpatient RCD surgery. After adjusting for age, sex, race, education, area deprivation, and body mass index, all O*NET variables considered were associated with RCD surgery (HR per point increase range=1.10–1.45, all P<0.005). A total of 100 929 people reported lifetime job histories, in which greater exposures were significantly associated with RCD surgery after >10 years of work (eg, HR for 11–20 versus 0 years with static strength score ≥4 = 2.06, 95% confidence interval 1.39–3.04).

**Conclusion:**

Workplace physical demands are an important risk factor for RCD surgery, particularly for workers with more than a decade of exposure.

Injury to the shoulder can be a substantial source of occupational disability, with shoulder pain impacting 18–26% of adults in prevalence surveys (1) and shoulder injuries leading to weeks away from work (in 2015, the US median days away from work due to shoulder injury was 23 days) (2). Rotator cuff disease (RCD) is the most common cause of shoulder disability with prevalence estimates for rotator cuff tears (both symptomatic and asymptomatic) in the general population consistently >10% in developed countries (3–6). Age is the predominant, well-established risk factor for development of a cuff tear (7, 8), but physical occupational demands may also play an important role in tear development, symptom onset, and symptom severity. Symptomatic cuff disease resulting in functional limitations is particularly concerning, especially for individuals who need to remain in the workforce. It is well established that frequency of rotator cuff surgery has been increasing in both the UK and the US (9, 10).

Occupations that require great upper extremity physical demands put individuals’ joints at greater risk of mechanical stresses that may lead to RCD. While evidence has accumulated demonstrating that occupational upper extremity loads are associated with risk of shoulder disorders generally (11–14), few studies have examined the specific relationship with RCD requiring surgery. A prospective study considering development and progression of rotator cuff tears demonstrated mixed results when evaluating associations with different measures of physical demands, but did not specifically measure occupational demands (15, 16). Several cross-sectional and retrospective studies have identified associations with work-related exposures and rotator cuff pathologies (13, 17–20), but these may be susceptible to healthy worker survivor bias. In addition, several of these studies relied on self-reported occupational exposures, potentially leading to differential exposure assessment among symptomatic workers (13, 18, 19). The purpose of this study was to examine the relationship between occupational exposures and the risk of symptomatic RCD requiring surgery in a population-based, prospective cohort with unbiased assessment of work exposures based on job titles.

## Methods

### UK Biobank population

Our study population was derived from the UK Biobank, a prospective cohort of half a million people registered with the National Health Service (21). Between 2006 and 2010, people 40–69 years of age were enrolled across the UK through 21 assessment centers (22). People living ≤25 miles from an assessment center were identified through the National Health Service patient register and mailed invitations to participate (>9 million invited) (23). The National Health Service Research Ethics Committee approved UK Biobank. The Washington University Institutional Review Board determined this study to be exempt from oversight. Participants gave informed consent and completed a touchscreen questionnaire including questions about demographics, pain at different anatomical locations that interfered with usual activities, self-reported medical history, and how frequently their current job involved heavy physical work.

### Occupational exposures

For participants who were employed at enrollment, trained UK Biobank staff collected information on current job title during a verbal interview and coded each job according to the UK Standard Occupational Classification (SOC) System 2000 (24). Between June and September of 2015, participants with an email address (N=324 653, including both those employed and retired/unemployed at enrollment), were contacted to collect lifetime job histories using a web-based tool that coded jobs into the UK SOC 2000 system (25). Our team linked UK SOC codes to the US SOC 2010 system in order to allow use of a recently developed job exposure matrix (JEM) based on the US Occupational Information Network (O*NET) database (26). Briefly, O*NET is a publicly available resource containing scores rating the physical and mental requirements of >800 jobs based on questionnaire responses from sampled workers and occupational experts (27). For the purposes of this study, we selected eight variables scored in O*NET as possibly relevant occupational exposures for the shoulder. Two of these variables estimate the level of a specific functional ability needed for job performance: static strength (ability to exert maximum muscle force to lift/push/pull/carry objects) and dynamic strength (ability to exert muscle force repeatedly or continuously over time). Two variables estimate the level of a particular work activity needed for job performance: handling & moving objects (using hands and arms in handling/installing/positioning/moving materials and manipulating things) and performing general physical activities (performing physical activities that require considerable use of your arms and legs and moving your whole body). Variables for abilities and work activities were measured on a 0–7 scale, with 7 representing the greatest level needed for job performance (examples of these levels are provided in the legend of figure 2). Finally, four variables estimate the amount of time spent under specific work conditions: time spent using your hands to handle/control/feel objects/tools/controls; time spent in cramped work space that requires awkward positions; time spent exposed to whole body vibration; and time spent making repetitive motions. Variables for work conditions were measured on a 1–5 scale, with 1 representing no time spent under specified conditions and 5 representing continual/almost continual time spent under specific conditions.

### Rotator cuff disease surgery ascertainment

National Health Service Hospital Episode Statistics (HES) records are linked to the UK Biobank providing information on diagnoses and procedures during hospital admissions between 2006 and 2020 (this included same-day and overnight admissions). HES records are coded using the International Classification of Diseases, 10^th^ revision (ICD-10) and procedures are coded using the Office of Population Censuses and Surveys Classification, 4^th^ revision. Our case definition for RCD surgery cases required both: (i) a diagnosis with an ICD-10 code of M75.1 or S46.0 made at a visit with a physician specializing in trauma/orthopedics, and (ii) a surgical procedure consistent with RCD treatment (supplementary material, www.sjweh.fi/article/4062, table S1). RCD surgical procedures included arthroscopic or open soft tissue procedures such as rotator cuff repair or sub-acromial decompression as well as joint arthroplasty when paired with a procedure code specific to rotator cuff pathology.

### Statistical analyses

As we were interested in incident symptomatic RCD, we excluded from analyses people reporting either tendonitis or ≥3 months of neck/shoulder pain at their baseline UK Biobank assessment visit, and people with a RCD diagnosis (ICD-10 of M75.1 or S46.0) from linked HES records prior to their UK Biobank enrollment date (based on data available back to 2006). Follow-up time for an incident RCD surgery started the date of an individual’s baseline assessment visit and ended at the first of: RCD surgery, death, loss-to-follow-up, or 31 December 2020 (the last date with available hospital data). As follow-up is obtained through linkage to hospital records, loss-to-follow-up only occurs when participants have reportedly left the country which has occurred in <0.03% of the population.

Initial analyses focused on estimating associations between characteristics of participants’ jobs reported at the baseline assessment visit and risk of incident RCD surgery. For this aim, we included people who reported a current job title at their baseline interview. A fraction of the population was retired, unemployed or had no job to report and were excluded. Additionally, we excluded people whose job code could not be linked to the O*NET job exposure matrix, specifically those with military jobs or non-specific job codes (eg, Transportation Workers, All Others). We then described distributions of each O*NET variable within the remaining population. Associations between each occupational exposure captured by O*NET and risk of RCD surgery were estimated using Cox regression adjusting for age at enrollment, sex, race, Townsend Deprivation Index (TDI, a neighborhood-level measure of deprivation), education, and body mass index (BMI) (adjustments were selected based on factors that could influence occupation that were also associated with RCD surgery in a prior UK Biobank study) (28). Participants with missing data for the included variables were excluded from regression models.

Immediate occupational effects on frequency of RCD surgery may be more likely to reflect differences in treatment-seeking behavior than differences in disease risk. To better isolate longer-term effects on disease risk we conducted sensitivity analyses with two-year, four-year, and ten-year time lags in which we only included RCD surgeries occurring two (and four and ten) years after the job title was reported at the baseline interview. Many of our O*NET measures were correlated with each other and with self-reported exposure to heavy physical work. To determine whether the O*NET variables were contributing uniquely predictive information, we also evaluated models that included the UK Biobank self-reported measure of heavy physical work.

Our second set of analyses focused on estimating associations between lifetime job histories and risk of RCD surgery. For this aim, we included people for whom a lifetime job history was available (including everyone, regardless of employment status at their baseline visit for the UK Biobank). We excluded people who had any job codes in their job history that could not be linked to the O*NET job exposure matrix (military occupations and non-specific jobs). While people could have job histories up until 2015, we only included time spent in jobs prior to their baseline visit so as not to include time occurring after a RCD surgery.

To calculate exposure duration across time we dichotomized the O*NET scores by creating whole number thresholds that identified people in approximately the top quartile of exposure for each variable as based on the distributions for jobs reported at baseline. For example, for the ‘Handling & Moving Objects’ measure, which has a score range of 0–7, time in a job with a score ≥4.0 was considered exposed. For our calculation of exposure duration, a job with work-weeks >30 hours was counted as full-time and the number of years in that job were summed to get a total number of exposed-years. A job with work-weeks of ≤30 hours counted as part-time and the number of years in that job were summed and divided by two to get a total number of exposed-years. We allowed a maximum of one exposure-year to be accumulated during any one calendar year, even if an individual reported working multiple jobs concurrently. Lifetime exposures for all variables were categorized as 0, 1–10, 11–20, 21–40, and ≥41 years. Associations with risk of RCD surgery were estimated using Cox regression, adjusting for age at enrollment, sex, race, TDI, education, and BMI.

## Results

### Associations with current job at baseline UK Biobank assessment visit

**Figure 1:**
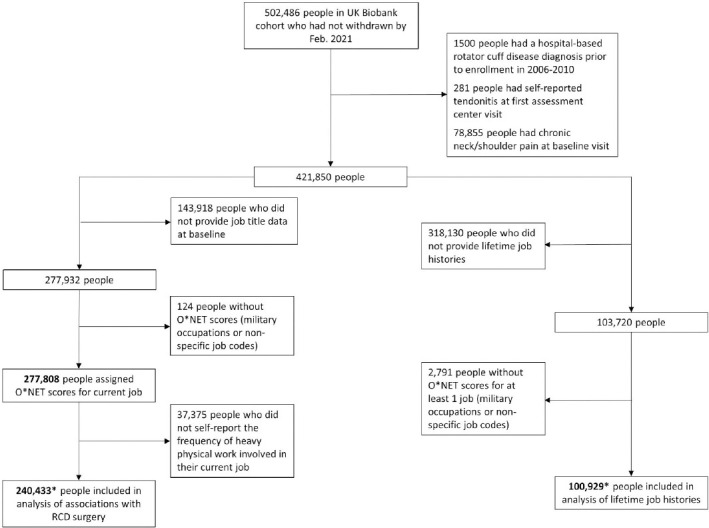
Selection of UK Biobank study populations with current job titles and lifetime job histories. *There were 63 614 participants with information on both current job title at baseline and lifetime job history.

We excluded 78 855 people reporting chronic neck/shoulder pain at baseline, 1500 people with a hospital-based RCD diagnosis prior to enrollment, and 281 people with self-reported tendonitis at their baseline UK Biobank visit, leaving 421 850 people without evidence of prevalent symptomatic disease (figure 1). Of these, 143 918 (34%) people did not provide job title data at baseline, and 124 (0.03%) people had jobs that could not be scored with O*NET. Among the remaining 277 808 people, the median age was 54 years, 48.6% were men, and 93.9% were white (table 1). Most people reported that their jobs never or rarely involved heavy manual/physical work, while 6.3% reported that their jobs always involved heavy manual/physical work. Across follow-up, 1997 RCD surgery cases were identified.

**Table 1:** Characteristics of UK Biobank population with current job titles at baseline enrollment visit and with reported lifetime job history. [IQR=interquartile range]

	Population with job title at enrollment		Population with lifetime job history	
	
	N (%)	Median (IQR)	N (%)	Median (IQR)
Total	277 808		100 929	
Age		54 (48, 60)		57 (50, 62)
Male sex	134 929 (48.57)		44 692 (44.28)	
Race				
White	260 906 (93.92)		98 236 (97.33)	
Black	4943 (1.78)		520 (0.52)	
Asian	6472 (2.33)		933 (0.92)	
Mixed	1747 (0.63)		455 (0.45)	
Unknown	3740 (1.35)		785 (0.78)	
Townsend deprivation index ^a^		-0.21 (-0.36, 0.04)		-0.25 (-0.39, -0.04)
Body mass index (BMI, kg/m^2^) ^b^				
Underweight (<18.5)	1326 (0.48)		597 (0.59)	
Healthy weight (18.5–<25.0)	94 049 (33.97)		39 614 (39.33)	
Overweight (25.0–<30.0)	117 795 (42.55)		41 553 (41.25)	
Obese (≥30.0)	63 701 (23.01)		18 968 (18.83)	
Job involves heavy manual or physical work ^c^				
Never/rarely	161 166 (65.82)		49 314 (76.6)	
Sometimes	51 955 (21.22)		10 638 (16.53)	
Usually	16 262 (6.64)		2600 (4.04)	
Always	15 483 (6.32)		1823 (2.83)	

a Townsend Deprivation Index missing for 361 people with job title at enrollment and 107 people with lifetime job history.

b BMI missing for 937 people with job title at enrollment and 197 people with lifetime job history.

c Information on heavy manual/physical work missing for 32 942 people with job title at enrollment and 36 554 people with lifetime job history.

**Figure 2:**
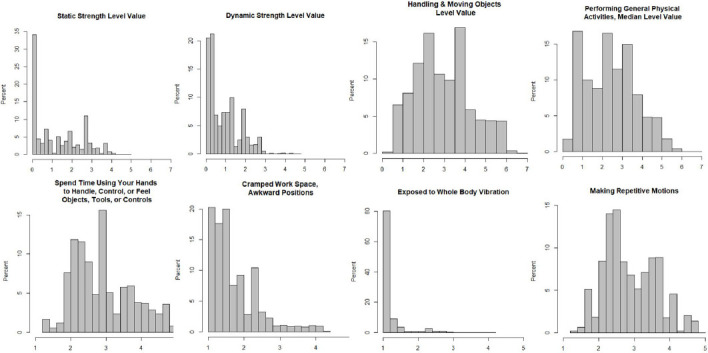
Distribution of O*NET variables among the 277 808 people in the UK Biobank with current job titles at baseline enrollment visit. For ‘Static Strength’, ‘Dynamic Strength’, ‘Handling and Moving Objects’, and ‘Performing General Physical Activities’, scores of 7 represent the greatest level of that ability or activity needed for job performance. For the evaluation of jobs, O*NET provides example tasks that correspond to different values, including for ‘Static Strength’ 1=Push an empty shopping cart and 6=Lift 75-pound bags of cement onto a truck; for ‘Dynamic Strength’ 2=Use pruning shears to trim a brush and 6=Perform a gymnastics routine using the rings; for ‘Handling and Moving Objects’ 2=Change settings on copy machines and 6=Load boxes on an assembly line; and for ‘Performing General Physical Activities’ 1=Walk between work stations in a small office and 6=Climb up and down poles to install electricity. For ‘Spend Time Using Your Hands to Handle, Control, or Feel Objects, Tools, or Controls’, ‘Cramped Work Space, Awkward Positions’, ‘Exposed to Whole Body Vibration’, and ‘Making Repetitive Motions’ scores of 1 represent no time spent under specified conditions and scores of 5 represent continual/almost continual time spent under specified conditions.

Distributions of physical exposures were evaluated based on O*NET scores linked to job titles. Scores for O*NET variables of ‘static strength’, ‘dynamic strength’, ‘cramped work space, awkward positions’, and ‘exposure to whole body vibration’ were notably right-skewed, with most people employed in jobs with low exposure (figure 2). The most extreme skew was observed for ‘exposure to whole body vibration’, for which nearly 50% of the population had jobs indicating no exposure. By comparison, scores for O*NET variables ‘handling & moving objects’, ‘performing general physical activities’, ‘spending time using your hands to handle/control/feel objects/tools/controls’, and ‘making repetitive motions’ were more evenly distributed across the range of possible values.

For analyses estimating the association of work exposures with RCD surgery we limited the population to the 240 433 people with O*NET scores, information on self-reported exposure to physical work, and information on potential confounders (age, sex, race, TDI, education and BMI) (figure 1). Among this population, there were 1623 cases of RCD surgery across a median follow-up time of 11.9 years (interquartile range: 11.2–12.6). In multivariable models adjusting for age, sex, race, TDI, education, and BMI, greater exposures to physical demands were associated with increased risk of RCD surgery as captured by all the O*NET variables with the exception of ‘making repetitive motions’ (table 2). The self-reported heavy physical work measure was also associated with increased risk of RCD surgery in multivariable analysis (table 2). Among the O*NET variables scored on a 1–5 scale, the strongest association was observed for exposure to whole body vibration, with each 1-point increase associated with 45% increased risk of RCD surgery [95% confidence interval (CI) 30–61%, table 2]. This was followed by exposure to cramped work space or awkward positions, for which each 1-point increase was associated with 25% increased risk of RCD surgery (95% CI 16–33%). Among the O*NET variables scored on a 0–7 scale, the strongest association was observed for dynamic strength, with each 1-point increase associated with 20% increased risk of RCD surgery (95% CI 13–28%), followed by performing general physical activities with each 1-point increase associated with 16% increased risk of RCD surgery (95% CI 12–21%).

**Table 2:** Associations between Occupational Information Network (O*NET) characteristics based on job title at baseline assessment and incidence of rotator cuff disease surgery among 240 433 people in the UK Biobank [HR=hazard ratio; HR_adj_=adjusted HR; CI=confidence interval].

Characteristic	Multivariable associations ^a^		Multivariable model with self-reported occupational manual labor ^a^	
	
	HR_adj_ (95% CI)	P-value	HR_adj_ (95% CI)	P-value
O*NET ^b^				
Static strength	1.13 (1.09–1.18)	<0.0001	1.03 (0.98–1.08)	0.2191
Dynamic strength	1.20 (1.13–1.28)	<0.0001	1.04 (0.97–1.11)	0.2660
Handling & moving objects	1.12 (1.08–1.16)	<0.0001	1.02 (0.98–1.07)	0.3185
Performing general physical activities	1.16 (1.12–1.21)	<0.0001	1.07 (1.02–1.12)	0.0063
Spend time using your hands to handle, control, or feel objects, tools, or controls	1.10 (1.04–1.17)	0.0014	1.00 (0.94–1.06)	0.9118
Cramped work space, awkward positions	1.25 (1.16–1.33)	<0.0001	1.09 (1.01–1.18)	0.0235
Exposed to whole body vibration	1.45 (1.30–1.61)	<0.0001	1.21 (1.07–1.36)	0.0018
Spend time making repetitive movements	0.99 (0.92–1.06)	0.6797	0.94 (0.88–1.01)	0.1089
UK Biobank self-reported measure				
Job involves heavy manual or physical work ^c^				
Never/rarely	Ref.	Ref.		
Sometimes	1.45 (1.29–1.64)	<0.0001		
Usually	1.94 (1.64–2.29)	<0.0001		
Always	2.12 (1.79–2.50)	<0.0001		

a Cox regression models adjusted for age, sex, race, Townsend Deprivation Index, education, and body mass index.

b Each O*NET characteristic was modeled per point increase on a 0–7 point scale for static strength; dynamic strength; handling & moving objects; and performing general physical activities; and modeled per point increase on a 1–5 point scale for spend time using your hands to handle, control, or feel objects, tools, or controls; cramped work space, awkward positions; and exposed to whole body vibration.

c Self-reported physical work values were based on the chosen response to the question “Does your work involve heavy manual or physical work?”

In sensitivity analyses, we found similar results when we evaluated associations between O*NET measures and RCD surgery after implementing 2-, 4-, and 10-year lags (supplementary table S2).

When associations between O*NET variables and RCD surgery were evaluated in multivariable models that included self-reported manual labor, statistically significant associations remained for general physical activities, exposure to cramped work space or awkward positions, and exposure to whole body vibration (all P<0.05, table 2). Self-reported heavy physical work was most strongly correlated with O*NET measures for handling and moving objects, static strength, and dynamic strength (supplementary table S3).

### Associations with lifetime job history

Of the 421 850 participants without evidence of prevalent disease, 103 720 reported a lifetime job history (figure 1). There were 2791 people (3%) with jobs that could not be scored with O*NET, leaving 100 929 people to include in analyses (figure 1, table 1). Among these people, 575 had subsequent RCD surgery. There were 63 614 people included in both the current job title and lifetime job history populations.

Among those reporting lifetime job history, we evaluated associations between time with a great exposure level (top quartile) and risk of RCD surgery for each O*NET measure after adjusting for age, sex, race, TDI, education, and BMI. A greater duration of time in jobs frequently requiring repetitive motions was not associated with RCD surgery risk. For all other O*NET variables evaluated, ≤10 years of exposure was not associated with a significant increase in RCD surgery risk, but >10 years of exposure was consistently associated with increased RCD surgery risk (table 3). Risk was highest in people with 11–40 years of great exposure for all variables. The strongest associations were observed for time with great static strength requirements [adjusted hazard ratio (HR_adj_) for 11–20 versus 0 years=2.06, 95% CI1.39– 3.04), performance of general physical activities (HR_adj_ for 11–20 versus 0 years=2.09, 95% CI 1.46–2.99), and exposure to whole body vibration (HR_adj_ for 11–20 versus 0 years=2.58, 95% CI 1.68–3.98).

**Table 3:** Associations between years in occupations with high scores for O*NET variables and rotator cuff disease surgery incidence among 100 895 people in the UK Biobank with lifetime job histories. [HR=hazard ratio; HR_adj_=adjusted HR; CI=confidence interval.]

Characteristic	Rotator cuff disease surgery N (%)		Multivariable associations ^a^	
	
	Yes (N=575)	No (N=100 354)	HR_adj_ (95% CI)	P-value
Static strength score ≥3.0 (years)				
0	452 (78.61)	85 613 (85.34)	Ref.	Ref.
1-10	42 (7.30)	7663 (7.64)	1.00 (0.73–1.38)	0.9894
11-20	28 (4.87)	2384 (2.38)	2.06 (1.39–3.04)	0.0003
21-40	37 (6.43)	3363 (3.35)	1.88 (1.32–2.67)	0.0005
≥41	16 (2.78)	1297 (1.29)	1.54 (0.91–2.60)	0.1093
Dynamic strength score ≥2.0 (years)				
0	400 (69.57)	77 793 (77.54)	Ref.	Ref.
1-10	69 (12.00)	12 456 (12.42)	1.08 (0.83–1.40)	0.5616
11-20	36 (6.26)	3539 (3.53)	1.91 (1.35–2.71)	0.0003
21-40	48 (8.35)	4676 (4.66)	1.86 (1.35–2.56)	0.0001
≥41	22 (3.83)	1856 (1.85)	1.57 (0.99–2.48)	0.0561
Handling & moving objects score ≥4.0 (years)				
0	338 (58.78)	65 970 (65.76)	Ref.	Ref.
1-10	90 (15.65)	17 241 (17.19)	1.02 (0.81–1.29)	0.8428
11-20	53 (9.22)	6183 (6.16)	1.58 (1.18–2.13)	0.0024
21-40	61 (10.61)	7931 (7.91)	1.36 (1.02–1.81)	0.0335
≥41	33 (5.74)	2995 (2.99)	1.38 (0.94–2.04)	0.1008
Performing general physical activities score ≥4.0 (years)				
0	425 (73.91)	82 220 (81.96)	Ref.	Ref.
1-10	46 (8.00)	8197 (8.17)	1.02 (0.75–1.40)	0.8854
11-20	34 (5.91)	2971 (2.96)	2.09 (1.46–2.99)	<.0001
21-40	50 (8.70)	4937 (4.92)	1.79 (1.31–2.45)	0.0003
≥41	20 (3.48)	1995 (1.99)	1.26 (0.78–2.03)	0.3479
Spend time using your hands to handle, control, or feel objects, tools, or controls score ≥4.0 (years)				
0	401 (69.74)	78 342 (78.09)	Ref.	Ref.
1-10	62 (10.78)	10 656 (10.62)	1.10 (0.84–1.45)	0.4921
11-20	36 (6.26)	4069 (4.06)	1.64 (1.16–2.33)	0.0053
21-40	50 (8.70)	5066 (5.05)	1.79 (1.31–2.45)	0.0003
≥41	26 (4.52)	2187 (2.18)	1.55 (1.01–2.38)	0.0457
Cramped work space, awkward positions score ≥3.0 (years)				
0	488 (84.87)	91 460 (91.17)	Ref.	Ref.
1-10	25 (4.35)	3839 (3.83)	1.03 (0.68–1.56)	0.8972
11-20	19 (3.30)	1564 (1.56)	1.98 (1.24–3.16)	0.0044
21-40	28 (4.87)	2337 (2.33)	1.89 (1.26–2.83)	0.0019
≥41	15 (2.61)	1120 (1.12)	1.60 (0.94–2.74)	0.0861
Exposed to whole body vibration score ≥2.0 (years)				
0	495 (86.09)	92 354 (92.06)	Ref.	Ref.
1-10	22 (3.83)	3589 (3.58)	1.01 (0.65–1.56)	0.9757
11-20	23 (4.00)	1388 (1.38)	2.58 (1.68–3.98)	<.0001
21-40	23 (4.00)	1925 (1.92)	1.95 (1.26–3.02)	0.0027
≥41	12 (2.09)	1064 (1.06)	1.32 (0.73–2.40)	0.3550
Spend time making repetitive motions score ≥4.0 (years)				
0	462 (80.35)	82 204 (81.94)	Ref.	Ref.
1-10	61 (10.61)	11 357 (11.32)	0.97 (0.74–1.27)	0.8147
11-20	23 (4.00)	3416 (3.41)	1.14 (0.74–1.76)	0.5599
21-40	23 (4.00)	2753 (2.74)	1.36 (0.88–2.10)	0.1650
≥41	6 (1.04)	590 (0.59)	1.28 (0.57–2.89)	0.5524

a Cox regression models adjusted for age, sex, race, Townsend Deprivation Index, education, and body mass index.

## Discussion

The need to better understand risk factors for symptomatic RCD requiring surgery cannot be understated. RCD is the most common cause of shoulder pain and a significant driver of time lost from work. A better understanding of occupational exposures can identify high risk groups and potentially guide preventative strategies. This large, population-based cohort study provided the opportunity for a unique, prospective examination of the associations between occupational exposures and surgical treatment for RCD. Our use of a JEM for estimating occupational exposures avoids the recall bias associated with self-report. This study demonstrated that, for all estimates of physical occupational exposures examined, there were significant associations between greater physical exposure and increased risk of rotator cuff surgery. All physical occupational exposures were positively correlated with one another to some degree, thus associations may reflect a combined effect of these exposures. Our O* NET JEM provided novel physical exposures of importance, not captured by the self-reported UK Biobank data, such as exposure to whole body vibration and exposure to cramped work space or awkward positions. Importantly, use of a JEM also provided estimates of exposures unaffected by participants’ disease status (29, 30). When examining lifetime job histories, we found the effects of all physical occupational exposures to be most evident among people with more than a decade of the greatest exposure levels.

Only a few prior studies have assessed associations between occupational exposures and RCD identified through radiographic or clinical examination. These studies have identified several risk factors, including arm elevation, heavy lifting, and use of handheld vibrating tools (13, 17–20). However, most have been retrospective or cross-sectional studies that could be susceptible to recall bias or selection bias. One recent prospective study of manufacturing and healthcare workers found that forceful hand exertions and certain upper arm postures were associated with development of rotator cuff syndrome (encompassing tears, tendinopathy, and subacromial pain syndrome) over a two-year follow-up period (31). Our study identified many similar risk factors, including heavy manual work, exposure to vibration, and awkward positions (which could include awkward upper arm postures).

Several studies from Denmark have also used a JEM to assess occupational shoulder burdens associated with surgery for shoulder impingement syndrome (which includes RCD surgeries along with other procedures) (12, 32, 33). A recent re-analysis of this cohort found that associations with occupational exposures were slightly stronger for cases with a rotator cuff syndrome diagnosis (M75.1) compared to other shoulder diagnoses such as tendinitis, impingement, and bursitis (34). Risk factors identified in these studies included forceful exertion, similar to our measures of static and dynamic strength, and hand-arm vibration, similar to our measure of whole body vibration (though our measure would be unlikely to capture many sources of vibration specific to the arm, such as use of power tools). Our study adjusted for several factors that could not be measured in the Danish studies, such as BMI and measures of socioeconomic status, and so the consistency of our findings further strengthens the evidence that these occupational risk factors are important contributors to RCD surgery risk.

One strength of our study was its assessment of associations between lifetime occupational exposures and RCD surgery. As participants in the UK Biobank represent an older population (40–69 years of age at enrollment), we would expect most rotator cuff tears in this population to have a degenerative component. For degenerative RCD, the accumulation of occupational exposures over time may be particularly important. In line with this hypothesis, it was only among people with over a decade of exposure to greater levels of physical demands that we observed significantly increased rates of RCD surgery. However, effects appeared to weaken among people with >40 years of exposure. This is likely due to a healthy worker survivor effect, in which people who are most susceptible to the adverse effects of heavy physical work are unlikely to remain in these occupations for many decades. Thus, workers with >40 years of exposure to physically demanding work may represent a select subgroup of people who are more resistant to the development of RCD. Similar patterns have been observed in occupational studies of carpal tunnel syndrome and lung cancer, in which the highest exposed workers did not have the highest rates of disease (35–37).

Our linkage with the O*NET JEM allows the use of unique and validated occupational measures not previously available in UK Biobank (26). This includes estimates for more specific exposures than those captured by self-report in UK Biobank. For instance, variables for exposure to whole body vibration and awkward positions were associated with RCD surgery independent of self-reported heavy physical work. A JEM can increase non-differential measurement error because all workers in the same job are assigned identical exposures, but JEM-estimated exposures are less susceptible to recall bias than self-reported exposures (29, 30). As a result, demonstrating associations between JEM-estimated exposures and RCD surgery strengthens the evidence that physical occupational exposures play an important role in RCD surgery risk. As O*NET was developed in the United States, some misclassification could occur due to differences in work duties in job classification in the UK. However, studies in UK Biobank and other European populations have shown that O*NET JEM estimates correspond well with other measures of physical work and appropriately identify the most and least physically-demanding jobs (26, 38, 39). Beyond the current study, the O*NET JEM could aid in future studies of other musculoskeletal diseases in the UK Biobank, particularly for the capture of lifetime exposures.

Our study had several limitations. First, jobs with the greatest physical demands may be difficult to perform with significant shoulder pain, leading to increased pursuit of surgical treatment. However, the associations we observed with job title at baseline were not attenuated in lagged analyses and associations with job exposure duration were strongest in those with more than a decade of exposure, indicating that our findings are not purely an artifact of acute treatment-seeking behaviors. Second, we lacked some specific exposure measures of importance for the rotator cuff. In particular, we could not capture prolonged arm elevation, which is one of the most consistently identified occupational risk factors for RCD and shoulder pain more broadly (40). Third, the UK Biobank population is more affluent than the UK as a whole and has been identified to have a healthy volunteer bias (23). The subset of participants who reported their lifetime job history appear to be even more affluent potentially because those doing computer-based work were more likely to have email and more easily able to complete the web-based questionnaire. As a result, people with the most physically-taxing work are likely under-represented in this study, and we expect absolute disease risks to be underestimates. However, if anything, this would reduce the chances of finding significant effects like those demonstrated here. Finally, by specifically evaluating cases managed surgically, we have likely focused on advanced or later-stage cuff disease. Although this likely provides a more precisely defined case group (excluding patients with other causes of shoulder pain), our case numbers will greatly underestimate the full burden of RCD.

In this large general population sample of the UK we observed numerous consistent associations between physical work exposures and RCD serious enough to require surgery. These associations were observed for a self-reported measure of heavy physical work, which captures variation at the individual level, and for several JEM-estimated physical work exposures, which are less likely to be influenced by reporting bias. Our results provide evidence that surgery for RCD may be considered as a potentially compensable occupational condition (41), particularly for people with more than a decade of work in physically-demanding occupations, where we observed a doubling of risk. Occupation should be recognized as an important contributor to the development of RCD, including degenerative RCD, which likely accounted for most of our cases given the age of our population. Workplace interventions could mitigate some of this risk, potentially increasing both worker health and productivity.

## Supplementary material

Supplementary material

## References

[1] Linaker CH, Walker-Bone K (2015). Shoulder disorders and occupation. Best Pract Res Clin Rheumatol.

[2] Bureau of Labor Statistics (2015). Nonfatal Occupational Injuries and Illnesses Requiring Days Away From Work.

[3] Yamamoto A, Takagishi K, Osawa T, Yanagawa T, Nakajima D, Shitara H (2010). Prevalence and risk factors of a rotator cuff tear in the general population. J Shoulder Elbow Surg.

[4] Liem D, Buschmann VE, Schmidt C, Gosheger G, Vogler T, Schulte TL (2014). The prevalence of rotator cuff tears:is the contralateral shoulder at risk?Am J Sports Med.

[5] Sher JS, Uribe JW, Posada A, Murphy BJ, Zlatkin MB (1995). Abnormal findings on magnetic resonance images of asymptomatic shoulders. J Bone Joint Surg Am.

[6] Tempelhof S, Rupp S, Seil R (1999). Age-related prevalence of rotator cuff tears in asymptomatic shoulders. J Shoulder Elbow Surg.

[7] Teunis T, Lubberts B, Reilly BT, Ring D (2014). A systematic review and pooled analysis of the prevalence of rotator cuff disease with increasing age. J Shoulder Elbow Surg.

[8] Yamaguchi K, Ditsios K, Middleton WD, Hildebolt CF, Galatz LM, Teefey SA (2006). The demographic and morphological features of rotator cuff disease. A comparison of asymptomatic and symptomatic shoulders. J Bone Joint Surg Am.

[9] Colvin AC, Egorova N, Harrison AK, Moskowitz A, Flatow EL (2012). National trends in rotator cuff repair. J Bone Joint Surg Am.

[10] Judge A, Murphy RJ, Maxwell R, Arden NK, Carr AJ (2014). Temporal trends and geographical variation in the use of subacromial decompression and rotator cuff repair of the shoulder in England. Bone Joint J.

[11] Miranda H, Punnett L, Viikari-Juntura E, Heliövaara M, Knekt P (2008). Physical work and chronic shoulder disorder Results of a prospective population-based study. Ann Rheum Dis.

[12] Svendsen SW, Dalbøge A, Andersen JH, Thomsen JF, Frost P (2013). Risk of surgery for subacromial impingement syndrome in relation to neck-shoulder complaints and occupational biomechanical exposures:a longitudinal study. Scand J Work Environ Health.

[13] Miranda H, Viikari-Juntura E, Heistaro S, Heliövaara M, Riihimäki H (2005). A population study on differences in the determinants of a specific shoulder disorder versus nonspecific shoulder pain without clinical findings. Am J Epidemiol.

[14] Seidler A, Romero Starke K, Freiberg A, Hegewald J, Nienhaus A, Bolm-Audorff U (2020). Dose-Response Relationship between Physical Workload and Specific Shoulder Diseases-A Systematic Review with Meta-Analysis. Int J Environ Res Public Health.

[15] Keener JD, Galatz LM, Teefey SA, Middleton WD, Steger-May K, Stobbs-Cucchi G (2015). A prospective evaluation of survivorship of asymptomatic degenerative rotator cuff tears. J Bone Joint Surg Am.

[16] Keener JD, Skelley NW, Stobbs-Cucchi G, Steger-May K, Chamberlain AM, Aleem AW (2017). Shoulder activity level and progression of degenerative cuff disease. J Shoulder Elbow Surg.

[17] Svendsen SW, Gelineck J, Mathiassen SE, Bonde JP, Frich LH, Stengaard-Pedersen K (2004). Work above shoulder level and degenerative alterations of the rotator cuff tendons:a magnetic resonance imaging study. Arthritis Rheum.

[18] Seidler A, Bolm-Audorff U, Petereit-Haack G, Ball E, Klupp M, Krauss N (2011). Work-related lesions of the supraspinatus tendon:a case-control study. Int Arch Occup Environ Health.

[19] Bodin J, Ha C, Petit Le Manac'h A, Sérazin C, Descatha A, Leclerc A (2012). Risk factors for incidence of rotator cuff syndrome in a large working population. Scand J Work Environ Health.

[20] Silverstein BA, Bao SS, Fan ZJ, Howard N, Smith C, Spielholz P (2008). Rotator cuff syndrome:personal, work-related psychosocial and physical load factors. J Occup Environ Med.

[21] Manolio TA, Weis BK, Cowie CC, Hoover RN, Hudson K, Kramer BS (2012). New models for large prospective studies:is there a better way?Am J Epidemiol.

[22] Collins R (2012). What makes UK Biobank special?Lancet.

[23] Fry A, Littlejohns TJ, Sudlow C, Doherty N, Adamska L, Sprosen T (2017). Comparison of Sociodemographic and Health-Related Characteristics of UK Biobank Participants With Those of the General Population. Am J Epidemiol.

[24] United Kingdom Office for National Statistics SOC (2000). Previous Version of the Standard Occupational Classification.

[25] De Matteis S, Jarvis D, Young H, Young A, Allen N, Potts J (2017). Occupational self-coding and automatic recording (OSCAR):a novel web-based tool to collect and code lifetime job histories in large population-based studies. Scand J Work Environ Health.

[26] Yanik EL, Stevens MJ, Harris EC, Walker-Bone KE, Dale AM, Ma Y (2022). Physical work exposure matrix for use in the UK Biobank. Occup Med (Lond).

[27] Cifuentes M, Boyer J, Lombardi DA, Punnett L (2010). Use of O*NET as a job exposure matrix:A literature review. Am J Ind Med.

[28] Yanik EL, Colditz GA, Wright RW, Saccone NL, Evanoff BA, Jain NB (2020). Risk factors for surgery due to rotator cuff disease in a population-based cohort. Bone Joint J.

[29] Viikari-Juntura E, Rauas S, Martikainen R, Kuosma E, Riihimäki H, Takala EP (1996). Validity of self-reported physical work load in epidemiologic studies on musculoskeletal disorders. Scand J Work Environ Health.

[30] Dale AM, Strickland J, Gardner B, Symanzik J, Evanoff BA (2010). Assessing agreement of self-reported and observed physical exposures of the upper extremity. Int J Occup Environ Health.

[31] Meyers AR, Wurzelbacher SJ, Krieg EF, Ramsey JG, Crombie K, Christianson AL (2021). Work-Related Risk Factors for Rotator Cuff Syndrome in a Prospective Study of Manufacturing and Healthcare Workers. Hum Factors.

[32] Dalbøge A, Frost P, Andersen JH, Svendsen SW (2014). Cumulative occupational shoulder exposures and surgery for subacromial impingement syndrome:a nationwide Danish cohort study. Occup Environ Med.

[33] Dalbøge A, Frost P, Andersen JH, Svendsen SW (2018). Surgery for subacromial impingement syndrome in relation to intensities of occupational mechanical exposures across 10-year exposure time windows. Occup Environ Med.

[34] Dalbøge A, Frost P, Andersen JH, Svendsen SW (2020). Exposure-response relationships between cumulative occupational shoulder exposures and different diagnoses related to surgery for subacromial impingement syndrome. Int Arch Occup Environ Health.

[35] Kapellusch JM, Gerr FE, Malloy EJ, Garg A, Harris-Adamson C, Bao SS (2014). Exposure-response relationships for the ACGIH threshold limit value for hand-activity level:results from a pooled data study of carpal tunnel syndrome. Scand J Work Environ Health.

[36] Applebaum KM, Malloy EJ, Eisen EA (2007). Reducing healthy worker survivor bias by restricting date of hire in a cohort study of Vermont granite workers. Occup Environ Med.

[37] Brown DM, Picciotto S, Costello S, Neophytou AM, Izano MA, Ferguson JM (2017). The Healthy Worker Survivor Effect:Target Parameters and Target Populations. Curr Environ Health Rep.

[38] Evanoff B, Yung M, Buckner-Petty S, Baca M, Andersen JH, Roquelaure Y (2019). Cross-national comparison of two general population job exposure matrices for physical work exposures. Occup Environ Med.

[39] Yung M, Evanoff BA, Buckner-Petty S, Roquelaure Y, Descatha A, Dale AM (2020). Applying two general population job exposure matrices to predict incident carpal tunnel syndrome:A cross-national approach to improve estimation of workplace physical exposures. Scand J Work Environ Health.

[40] Wærsted M, Koch M, Veiersted KB (2020). Work above shoulder level and shoulder complaints:a systematic review. Int Arch Occup Environ Health.

[41] Industrial ndustries Advisory Council Guidance on evaluating evidence on health risks associated with occupational exposures.

