# Supplementary material for: Occupational demands associated with rotator cuff disease surgery in the UK Biobank
Source: Scand J Work Environ Health. 2022 Dec 30;49(1):53–63. doi: 10.5271/sjweh.4062 (PMC10549913; doi:10.5271/sjweh.4062)
Supplement: Supplementary material [file SJWEH-49-53-S001.pdf]

Occupational demands associated with rotator cuff disease surgery in the UK Biobank[1]

by Elizabeth L Yanik, PhD, ScM,[2] Jay D Keener, MD, Martin J Stevens, PhD, Karen E Walker-Bone, PhD, Ann Marie Dale, PhD, Yinjiao Ma, MPH, Graham A Colditz, MD, DrPH, Rick W Wright, MD, Nancy L Saccone, PhD, Nitin B Jain, MD, MSPH, Bradley A Evanoff, MD, MPH

1. Supplementary material
2. Correspondence to: Elizabeth L. Yanik, PhD ScM, Department of Orthopaedic Surgery, Washington University School of Medicine, 660 S. Euclid Ave, Campus Box 8233, St. Louis, MO 63110, USA. [E-mail: yanike@wustl.edu]

Table S1: Procedure codes used to define rotator cuff surgery when paired with an ICD-10 code of M75.1 or S46.0

Category of Surgical Treatment	OPCS-4 code	Only included if accompanied by an OPCS-4 code indicating a procedure done on the shoulder? [a]
Rotator Cuff Repair	T791, T793, T794, T795	No
Subacromial Decompression	O291	No
	W844	Yes
Shoulder Arthroplasty	O061, O062, O071, O108, W491, W494, W504, W511, W961, W962, W965, W971, W972, W973, W975, W981, W986	No
	W551, W562, W572, W581	Yes
Unspecified Arthroscopic Procedures of the Shoulder	W848, W849, W868, Y767	Yes

[a]Shoulder codes: Z542, Z813, Z814, Z891

ICD-10=International Classification of Diseases, 10th revision

OPCS-4=OPCS Classification of Interventions and Procedures version 4

Supplementary Table S2: Associations between O*NET characteristics based on job title at baseline assessment and incidence of rotator cuff disease surgery with different period of exposure lagging among 239,591 people with ≥2 years of follow-up and 238,055 people with ≥4 years of follow-up, and 231,897 people with ≥10 years of follow-up in the UK Biobank

	Multivariable associations with 2-year lag[a] (N=239,591)		Multivariable associations with 4-year lag[a] (N=238,055)		Multivariable associations with 10-year lag[a] (N=231,897)	
**O*NET Characteristic[b]**	**aHR (95% CI)**	**p-value**	**aHR (95% CI)**	**p-value**	**aHR (95% CI)**	**p-value**
Static Strength	1.13 (1.08, 1.18)	<0.0001	1.11 (1.06, 1.16)	<0.0001	1.10 (0.98, 1.24)	0.0966
Dynamic Strength	1.20 (1.13, 1.28)	<0.0001	1.18 (1.10, 1.27)	<0.0001	1.20 (1.01, 1.43)	0.0348
Handling & Moving Objects	1.12 (1.08, 1.16)	<0.0001	1.10 (1.05, 1.15)	<0.0001	1.12 (1.01, 1.25)	0.0312
Performing General Physical Activities	1.16 (1.11, 1.21)	<0.0001	1.15 (1.09, 1.20)	<0.0001	1.14 (1.01, 1.28)	0.0297
Spend Time Using Your Hands to Handle, Control, or Feel Objects, Tools, or Controls	1.10 (1.03, 1.17)	0.0033	1.08 (1.01, 1.16)	0.0315	1.13 (0.96, 1.34)	0.1466
Cramped Work Space, Awkward Positions	1.24 (1.15, 1.33)	<0.0001	1.22 (1.13, 1.32)	<0.0001	1.24 (1.02, 1.51)	0.0307
Exposed to Whole Body Vibration	1.42 (1.27, 1.59)	<0.0001	1.40 (1.23, 1.59)	<0.0001	1.56 (1.15, 2.11)	0.0039
Spend Time Making Repetitive Motions	0.98 (0.91, 1.06)	0.6757	0.96 (0.88, 1.04)	0.2814	1.04 (0.85, 1.27)	0.7096

[a]Cox regression models adjusted for age, sex, race, Townsend Deprivation Index, education, and body mass index. Models with a 2-year lag included 1485 cases. Models with a 4-year lag included 1193 cases. Models with a 10-year lag included 206 cases.

[b]Each O*NET characteristic was modeled per point increase on a 0-7 point scale for static strength; dynamic strength; handling & moving objects; and performing general physical activities; and modeled per point increase on a 1-5 point scale for spend time using your hands to handle, control, or feel objects, tools, or controls; cramped work space, awkward positions; and exposed to whole body vibration.

aHR=adjusted hazard ratio, O*NET=Occupational Information Network

Supplemental Table S3: Correlation matrix between all physical work measures showing Spearman correlation coefficients

	UKB Self-Reported Heavy Manual/ Physical Work	O*NET Static Strength	O*NET Dynamic Strength	O*NET Handling & Moving Objects	O*NET Performing General Physical Activities	O*NET Spend Time Using Your Hands to Handle, Control, or Feel Objects, Tools, or Controls	O*NET Cramped Work Space, Awkward Positions	O*NET Exposed to Whole Body Vibration	O*NET Spend Time Making Repetitive Motions
UKB Self-Reported Heavy Manual/Physical Work	1								
O*NET Static Strength	0.50	1							
O*NET Dynamic Strength	0.49	0.91	1						
O*NET Handling & Moving Objects	0.50	0.87	0.81	1					
O*NET Performing General Physical Activities	0.49	0.87	0.83	0.91	1				
O*NET Spend Time Using Your Hands to Handle, Control, or Feel Objects, Tools, or Controls	0.32	0.50	0.58	0.58	0.43	1			
O*NET Cramped Work Space, Awkward Positions	0.39	0.72	0.69	0.69	0.75	0.50	1		
O*NET Exposed to Whole Body Vibration	0.31	0.53	0.48	0.52	0.62	0.27	0.66	1	
O*NET Spend Time Making Repetitive Motions	0.16	0.23	0.39	0.27	0.10	0.73	0.19	0.02	1

UKB Self-reported heavy manual/physical work was coded as: Never/rarely=0, Sometimes=1, Usually=2, Always=3

O*NET=Occupational Information Network
